# Clinical experiences and learning curves from robot-assisted neurosurgical biopsies with Stealth Autoguide™

**DOI:** 10.1093/noajnl/vdae079

**Published:** 2024-05-14

**Authors:** Johan Ljungqvist, Hanna Barchéus, Fatima Abbas, Anneli Ozanne, Daniel Nilsson, Alba Corell

**Affiliations:** Institute of Neuroscience and Physiology, Department of Clinical Neuroscience, University of Gothenburg, Sahlgrenska Academy, Gothenburg, Sweden; Department of Neurosurgery, Sahlgrenska University Hospital, Gothenburg, Sweden; Institute of Neuroscience and Physiology, Department of Clinical Neuroscience, University of Gothenburg, Sahlgrenska Academy, Gothenburg, Sweden; Institute of Neuroscience and Physiology, Department of Clinical Neuroscience, University of Gothenburg, Sahlgrenska Academy, Gothenburg, Sweden; Institute of Health and Care Sciences, Sahlgrenska Academy, University of Gothenburg, Gothenburg, Sweden; Institute of Neuroscience and Physiology, Department of Clinical Neuroscience, University of Gothenburg, Sahlgrenska Academy, Gothenburg, Sweden; Department of Neurosurgery, Sahlgrenska University Hospital, Gothenburg, Sweden; Institute of Neuroscience and Physiology, Department of Clinical Neuroscience, University of Gothenburg, Sahlgrenska Academy, Gothenburg, Sweden; Department of Neurosurgery, Sahlgrenska University Hospital, Gothenburg, Sweden

**Keywords:** brain tumors, biopsy, glioma, neurosurgery, neurosurgical procedure

## Abstract

**Background:**

Biopsies of intracranial lesions are a cornerstone in the diagnosis of unresectable tumors to guide neurooncological treatment; however, the procedure is also associated with risks. The results from the cranial robot guidance system Stealth Autoguide™ were studied after introduction at a neurosurgical department. Primary aims include the presentation of clinical and radiological data, accuracy of radiological diagnosis, learning curves of the new technology, diagnostic yield, and precision. The secondary aim was to study complications.

**Methods:**

Retrospective data inclusion was performed on patients ≥ 18 years undergoing biopsy with Stealth Autoguide™ due to suspected brain tumors in the first 3 years after the introduction of the technique. Data regarding clinical characteristics, intraoperative variables, pathological diagnosis, and complications were recorded. Analyses of learning curves were performed.

**Results:**

A total of 79 procedures were performed on 78 patients with a mean age of 62 years (SD 12.7, range 23–82), 30.8% were female. Tumors were often multifocal (63.3%) and supratentorial (89.9%). The diagnostic yield was 87.3%. The first-hand radiological diagnosis was correct in 62.0%. A slight decrease in operation time was observed, although not significant. The surgeon contributed to 12% of the variability.

**Conclusions:**

Robot-assisted biopsies with Stealth Autoguide™ seem to be comparable, with regards to complications, to frame-based and other frameless neurosurgical biopsies. Learning curves demonstrated no statistical differences in time of surgery and only 12% surgeon-related variation (ie, variation caused by the change of performing surgeon), suggesting a successful implementation of this technical adjunct.

## Lay Summary

Neurosurgeons will sometimes biopsy a tumor to officially diagnose it if it is too risky to remove with surgery. Biopsies are typically performed by the surgeon without the use of robots. In this study, the authors wanted to describe their experience with robot-assisted brain tumor biopsies. To do this, they reviewed the medical records of patients who had brain tumor biopsies done with a commercial robot. Their results showed that 78 patients had a robot-assisted brain tumor biopsy at their hospital. A diagnosis was achievable for 87% of these patients. The time taken for surgery did not seem to change much over the study period, and the surgeon only accounted for a small portion of the differences in the length of surgery. Although 41% of patients had some bleeding at the site of biopsy, the bleedings were most often small and rarely caused problems for the patient.

**Key Points**

- The rate of complication was comparable to previous literature.
- The diagnostic yield was 87.3%.
- The surgeon contributed to 12% variability; the rest was attributed to other factors.

**Importance of the Study**

This study presents the results after implementing the cranial robot guidance system Stealth Autoguide™ in clinical praxis. We analyzed possible learning curves after implementation and found a slight decrease in time during the study period, although without statistical significance. This finding, in addition to only 12% of the variability by the surgeon, suggests a successful implementation. The postoperative complications were comparable to previous studies on both frame-based and frameless studies. The diagnostic yield was 87.3%. Furthermore, in this study, we also identified some important pitfalls when performing intracranial biopsies. Neurosurgeons must be cautious when performing biopsies of lesions in the posterior fossa, especially very small targets, and when planning biopsies of suspected CNS lymphomas.

Neurosurgical biopsies play a crucial role in the histopathological diagnoses of intracranial lesions where surgical resection is deemed inappropriate.^1^ Once a histopathological diagnosis has been established, further neurooncological treatment can be initiated.^2^ Various neurosurgical methods are available for the retrieval of biopsies. Historically, the traditional frame-based stereotactic biopsy has been the most frequently utilized method of choice in high-precision procedures, although the frameless stereotactic systems are increasingly gaining more popularity.^3–5^ The diagnostic yield between these 2 methods appears equivalent when comparing the Remebot® robot-assisted frameless surgery to the SINO surgical robot.^6–8^ Additionally, the study by Mallereau et al. studied the diagnostic rate in previous literature and found the inconclusive biopsies to be in a range of 2.6%–11.1% in frameless biopsies compared to 0.7–15.7 in frame-based procedures.^9^

Recently, the cranial robot guidance system Stealth Autoguide™ by Medtronic was introduced as an adjunct in the neurosurgical repertoire.^10^ The guidance system has been used, not only for biopsies, but also in other neurosurgical procedures requiring high precision such as for placement of stereotactic electroencephalography depth electrodes (SEEG) in patients with epilepsy, as well as for robot-assisted laser ablation.^11,12^ Due to the limited number of patients it’s difficult to conduct large randomized trials. Additionally, with new techniques that are not as resource-demanding and easier to use than the traditional frame-based procedure, more surgeons can be trained. These techniques are therefore gradually introduced in clinical praxis. The learning curve for neurosurgical techniques has previously been presented, but demonstrates a great heterogeneity in the assessed parameters.^13^ Furthermore, robot-assisted biopsies in neurosurgery have been studied, although to a lesser extent with regard to learning aspects.^9,14^ The Stealth Autoguide™ robot-assisted system has been scarcely studied in previous clinical trials with regard to biopsy procedures. This limits the predictive value; thus, it is important to conduct a clinical evaluation.

Complications previously reported in studies on stereotactic biopsies pose inherent challenges for comparison. These challenges arise from inconsistent reporting systems, variations in individual interpretations of complications, and insufficient information regarding postoperative hematomas and symptoms related to tumor progression.^4,15^ Postoperative complications from robot-assisted frameless biopsies have been reported to a lesser extent.^6,8,16^

This study aimed to investigate if there was a learning curve and to examine the diagnostic yield after the introduction of the Stealth Autoguide™ system in the biopsies of suspected brain tumors. The primary aims included a presentation of clinical and radiological characteristics as well as the accuracy of radiological diagnoses, learning curves of this new technology introduced in clinical practice, and diagnostic yield. Furthermore, as a secondary objective, we aimed to study complications, focusing on postoperative hematomas.

## Materials and Methods

### Study Population

The neurosurgical department at Sahlgrenska University Hospital is responsible for the neurosurgical care for approximately 1.8 million inhabitants in the Western region of Sweden. The cranial robot guidance system Stealth Autoguide™ was introduced in May 2020. All patients with newly diagnosed suspected primary intracranial intra-axial tumors are managed by the multidisciplinary team (MDT) at Sahlgrenska University Hospital. Patients were selected for biopsy through the MDT on a continuous basis as part of their clinical treatment. Patients with deep-seated lesions, such as those located in basal ganglia, were selected for stereotactic frame-based biopsy, otherwise, the patients have opted for robot-assisted biopsy if the trained surgeon was on duty.

The patient selection for undergoing intracranial biopsy included those whose tumors were deemed not resectable due to eloquence, patients with low-performance status but still would benefit from oncological therapy, and patients with unclear lesions on magnetic resonance imaging (MRI) in need of diagnosis to further guide treatment.

### Workflow

Patients selected for robot-assisted biopsies were admitted, and their MRI scans were imported to the workstation. The target and trajectory were planned the day before surgery, and caution was taken to avoid vessels. On the day of surgery, the images and the planned trajectory were transferred to the operating theater, and the images were registered to the skin of the patient. An incision was made, and a burr hole was placed in line with the trajectory using neuronavigation. The meninges were opened and the Stealth Autoguide™ steered the working channel of the biopsy needle to the target. A biopsy needle was then inserted, and biopsies were taken from the target and possibly at different depths in the trajectory line, when deemed appropriate. For small lesions, particularly in the posterior fossa, the O-arm was used to improve the accuracy of registration.

### Clinical, Radiological, and Surgical Variables

The following variables were studied: Age, gender, surgery date, which neurosurgeon performed the surgery, comorbidities, survival, symptoms at presentation, time of surgery, total time of anesthesia, complications according to Landriel-Ibanez classification system^17^ within 30 days after surgery, and histopathological diagnosis. Precision was assessed in the inconclusive cases with regard to the biopsy needle canal or postoperative air bubbles in the biopsy area.

The MRI scans assessed were performed at different hospitals in the region depending on where the patient was diagnosed. Therefore, MRI systems for image acquisition included 1.5T in addition to 3.0T scanners from various vendors (GE Healthcare, US; Philips, The Netherlands; Siemens Healthcare, Germany). Routinely, all MRI examinations included T1 weighted sequences with and without gadolinium contrast, T2 weighted sequences, and FLAIR sequences.

Radiological variables included lateralization, eventual multifocality, infra/supratentorial, main location, and size. From the radiological statement, the primary suspected diagnosis was collected, in addition to other suspected differential diagnoses, if stated. The radiological statement was primarily recorded from a neuroradiological assessment, which was performed during the MDT conference.

The time of surgery was calculated from knife incision to end of surgery (closure of wound). If the time of incision was missing, the mean preparation time for the same procedures was calculated from the start of anesthesia to the skin incision and then subtracted from the total procedure time. The experience of the surgeon performing the procedure was recorded (neurosurgery consultant or senior resident with a consultant colleague), as well as the number of tissue samples collected.

A postoperative head CT scan without contrast was routinely performed 6 hours after surgery as part of the clinical routine to detect postoperative hematoma. These CT scans were assessed by a clinical radiologist, in addition to a neurosurgery consultant (AC).

### Histopathological Diagnosis

The tissue samples from the biopsies were analyzed in clinical practice according to the World Health Organization (WHO) classification system of tumors in the central nervous system from the 2016 classification system up until the introduction of the updated WHO 2021 version.^18,19^ The diagnostic yield was analyzed. The samples were classified as inconclusive if;

1. No material was collected at all or if the surgery was disrupted due to complication or adverse event;
2. Material collected but:
  a. Classified as normal brain tissue;
  b. Was collected from the peripheral part of the lesion where a diagnosis could not be made, eg, border zone of a suspected glioblastoma or lymphoma treated with steroids, or;
  c. Tissue from the lesion was adequately retrieved but no diagnosis could be made due to necrotic area or cystic fluid not able to diagnosed.

### Statistical Analysis

Analyses were made using SPSS, version 29.0.2.0 (Chicago, IL, USA) and R Version 4.3.1.

VCA package 1.4.5. The statistical significance level was set to *P* < .05 and all tests were 2-sided. Central tendencies were presented as mean with standard deviation (SD), or median and interquartile range (IQR) if skewed. Other categorical data were analyzed with Pearson’s chi-square test. Independent sample *t*-test or Mann–Whitney U test were used when appropriate based on data type. For the surgeons with at least 10 surgeries, GLM (General Linear Model) analysis was performed using surgery order as the independent variable. Furthermore, a variance component analysis was performed to estimate the contribution of the surgeon as compared to error repeatability. The 15 first procedures for surgeons with at least 5 procedures (5 surgeons) were selected for analysis. The general linear model estimates the knife time for the procedures given the number of procedures performed. Furthermore, for the selected surgeons, the difference between the first and the following procedures are compared, ie, the procedure number is restricted to the First and Following. For this analysis, the variance components for the knife time are evaluated using First (First/Following) and Surgeon (5 selected surgeons) as factors in the model.

### Ethical Statement

This project was approved by the Ethics Review Authority (DNR 2022-00160-01). Patients included in this study approved inclusion through written informed consent.

## Results

In total, 79 biopsy procedures were performed in 78 patients during the study period. The mean age in the cohort was 62 years (SD 12.7 years, range 23–82 years), and 24 patients (30.8%) were female. Common comorbidities included hypertension (34.6%), other malignancies (17.9%), hyperlipidemia (16.7%), and cardiac disease (15.4%). Presenting symptoms included cognitive impairment (43.6%), motor deficits (39.7%), headache/vomiting (30.8%) and seizures (28.2%). Visual deficits, disorientation, and vertigo were present in approximately 20% of patients (20.5%, 17.9%, and 20.5%, respectively). See Table 1 for more data regarding clinical variables.

**Table 1.** Clinical Characteristics of the Patient Cohort (*n* = 78)

Variables	*N* = 78
Age, mean years (SD, range)	62 (12.7, 23-82)
Female, *n* (%)	24 (30.8)
*Comorbidities* ^1^	
Previously healthy, *n* (%)	26 (33.3)
Hypertension, *n* (%)	27 (34.6)
Other malignancy, *n* (%)	14 (17.9)
Hyperlipidemia, *n* (%)	13 (16.7)
Cardiac disease, *n* (%)	12 (15.4)
Diabetes, *n* (%)	9 (11.5)
Hypothyroidism, *n* (%)	4 (5.1)
COPD*, *n* (%)	2 (2.6)
Other**, *n* (%)	35 (44.9)
*Symptoms at presentation* ^2^	
Cognitive impairment, *n* (%)	34 (43.6)
Motor deficit, *n* (%)	31 (39.7)
Headache/vomiting, *n* (%)	24 (30.8)
Seizures, *n* (%)	22 (28.2)
Visual deficit, *n* (%)	16 (20.5)
Disorientation/confusion, *n* (%)	14 (17.9)
Vertigo, *n* (%)	16 (20.5)
Language deficit/disturbance, *n* (%)	9 (11.5)
Asymptomatic, *n* (%)	1 (1.3)

^1^More than one comorbidity could occur in 1 patient.

*Chronic obstructive pulmonary disease (COPD).

**Including nephropathy, osteoporosis, Chron’s disease, alcohol abuse, psychiatric diagnoses, kidney stones, psoriasis, allergies, and asthma.

^2^More than one symptom could occur in one patient.

### Radiological Variables and Accuracy of Radiological Diagnosis

The majority of tumors were multifocal (63.3%), supratentorial (89.9%) and were relatively equally distributed between temporal (25.3%), frontal (25.3%), and parietal lobes (19.0%). Most of the tumors were smaller than 4 cm (57.0%) and a third was between 4 and 6 cm (34.2%), see Table 2. The first-hand radiological diagnosis stated by the neuroradiologist on the radiology assessment was correct in 62.0% and the histological diagnosis was consistent with one of the suggested radiological diagnoses in 15.2%. Furthermore, the radiological diagnosis was incorrect in 10.1%.

**Table 2.** Radiological and Histopathological Characteristics of Tumor in Procedures (*n* = 79)

Variables	*N* = 79
Right side main, *n* (%)	42 (53.2)
Bilateral, *n* (%)	28 (35.4)
Multifocal, *n* (%)	50 (63.3)
Supratentorial, *n* (%)	71 (89.9)
Main temporal, *n* (%)	20 (25.3)
Main frontal, *n* (%)	20 (25.3)
Main parietal, *n* (%)	15 (19.0)
Main occipital, *n* (%)	7 (8.9)
Main basal ganglia/deep, *n* (%)	8 (10.0)
Main insular, *n* (%)	1 (1.2)
Main cerebellar, *n* (%)	6 (7.6)
Main brainstem, *n* (%)	2 (2.6)
*Size (diameter)*	
Smaller than 4 cm, *n* (%)	45 (57.0)
4–6 cm, *n* (%)	27 (34.2)
Larger than 6 cm, *n* (%)	7 (8.9)
*Primary suspected radiological diagnosis*	
First-hand radiological diagnosis correct, *n* (%)	49 (62.0)
Diagnosis among suggested, *n* (%)	12 (15.2)
Incorrect radiological diagnosis, *n* (%)	8 (10.1)
*Histopathological diagnosis*	
Glioblastoma, *n* (%)	46 (58.2)
Diffuse large B-cell lymphoma, *n* (%)	10 (12.7)
Astrocytoma grade 2 and 3, *n* (%)	6 (7.5)
Inflammation, *n* (%)	3 (3.8)
Metastasis^1^, *n* (%)	2 (2.5)
Astrocytoma, pilocytic grade 1, *n* (%)	1 (1.3)
Dysembryoplastic neuroepithelial tumor (DNET), *n* (%)	1 (1.3)
Inconclusive^2^, *n* (%)	10 (12.7)

^1^Including metastasis from malignant melanoma (*n* = 1) and T-cell lymphoma (*n* = 1).

^2^Including healthy brain tissue and signs of corticosteroid-treated DLBCL.

### Surgical Variables and Learning Curve

The mean time of surgery was 51 minutes (SD 22.4, range 13–113 minutes). Duration of surgery was slightly shorter in the procedures with patients in the supine position (*n* = 67, mean 50 minutes, SD 22.5) compared to the prone position (*n* = 12, 55 minutes, SD 22.6); however, this was not statistically significant (*P* = .44). The number of biopsies varied between 1 and 6 with a median of 4 biopsies at the target of trajectory or shallower. In 11 patients (13.8%) the number of biopsies was unspecified; however, in these cases, multiple biopsies were always retrieved.

For the specified number of biopsies, the total mean time of surgery was 60.5 minutes (SD 33.5) in the group with 1–2 biopsies, 51.0 minutes (SD 21.8) for 3–4 biopsies and 42.8 minutes (SD 21.1) in the group of 5 or more. Furthermore, there was no statistically significant difference when comparing multiple biopsies (5 or more) to fewer (< 5; *P* = .086).

**Figure 1.**
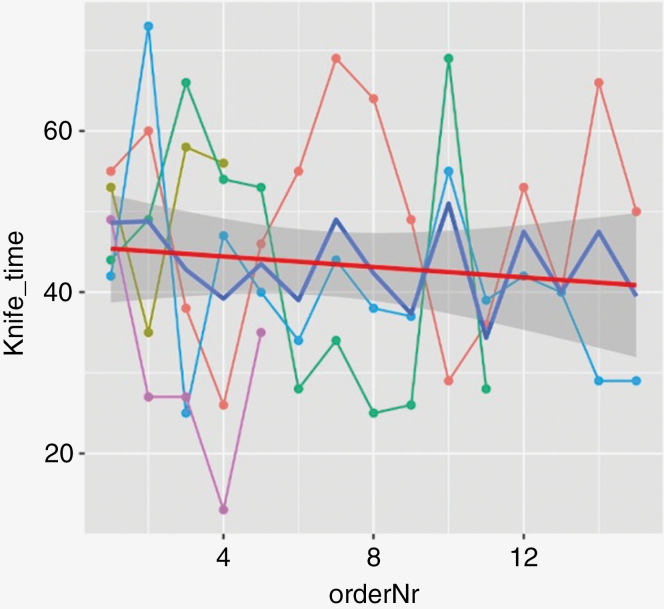
Operation time for the consecutive surgeries with colored lines representing the 5 most frequent individual neurosurgeons and the thicker inclined horizontal line representing the meantime (*n* = 61). The *x*-axis represents consecutive numbers of surgeries, and the *y*-axis represents the total operation time (knife time).

When statistically analyzing the possible learning curves, the 2 cases with an intraoperative CT scan were excluded (*n* = 2). There was a slight decrease in operation time over time from the introduction which indicates a learning curve. However, this difference was not significant, see Figure 1 with the red line indicating meantime. Furthermore, in a variance component analysis, the surgeon accounted for 12% of the variation in the time of surgery in the mixed model. The other percent of the variation resulted from other factors.

### Diagnostic Yield

In all 79 cases, tissue was sent for histopathological and molecular analysis when suitable. A histopathological diagnosis was achievable in 87.3% of cases. The remaining 10 cases (12.7%) were deemed inconclusive. For these patients, further clinical decisions were based primarily on radiological diagnosis in an MDC, which led to oncological treatment in 6 of the 10 inconclusive cases (in all 6 cases patients received temozolomide with additional radiotherapy in 2 patients). Four patients received no further treatment. However, in one patient an additional attempt at tissue sampling was carried out a few weeks later, leading to a conclusive histopathological diagnosis.

The majority of the inconclusive cases demonstrated intense or nodular contrast enhancement on preoperative radiology (60%), whereas 30% displayed mild or patchy enhancement and one case had no contrast enhancement whatsoever, see Table 3. There was no statistical significance. Six patients had pathologies smaller than 4 cm (60%). Furthermore, in 3 of the 10 patients, the findings were considered inconclusive; however, there was a clinical and radiological suspicion of a steroid-treated lymphoma, but the histopathological assessment was insufficient in establishing this diagnosis with certainty. Amongst the patients with inconclusive cases, 3 harbored infratentorial pathologies; 2 was located in the cerebellum and one was located in the brainstem. For supratentorial biopsies, the rate of conclusive biopsies was 90.1% and for infratentorial biopsies, it was 62.5% (*P* = .059). The inconclusive biopsies were evenly distributed over the study period.

**Table 3.** Crosstab Over Histopathological Results in Relation to Contrast Enhancement on Operative Magnetic Resonance Imaging (*n* = 79)

	Conclusive (*n* = 69)	Inconclusive (*n* = 10)	*P*-value
No contrast, *n* (%)	3 (4.3)	1 (10.0)	.43
Mild contrast, *n* (%)	9 (13.0)	3 (30.9)	.17
Intense/nodular contrast, *n* (%)	57 (82.6)	6 (60.0)	.11

When studying the postoperative CT scan in terms of precision, the biopsy needle canal or postoperative air bubbles were assessed. Four of the biopsies seem to have been too shallow compared to preoperative MRI. In 2 cases with suspected lymphoma, the preoperative MRI was performed 6 and 9 days preoperatively followed by high-dose steroid treatment. In one patient with a suspected lymphoma, the patient had undergone steroid treatment and the lesion had shrunk and the biopsy locally seemed to be in lesion, but a conclusive diagnosis could not be made (an increased number of inflammatory cells were noticed). In one patient the biopsy local was in the lesion but a final diagnosis could not be made due to too little biopsy material (clinically treated as a suspected lower-grade glioma). Finally, for 2 tumors in the cerebellum, the biopsy location seems to be slightly off target, but the contrast-enhancing lesions were very small in one case (5 mm) and the other without contrast enhancement (9 mm). Positron emission tomography (PET) was performed in 2 cases of a total of 10 inconclusive cases.

### Complications

Complications after surgery are presented in Table 4. The majority of patients (70.9%) suffered no postoperative complications. One patient suffered from postoperative leakage of cerebrospinal fluid and needed suture without anesthesia (grade 2a). In total 3 patients (3.8%) died within 30 days of surgery. Causes of death included epilepsy and lung embolism at day 14 in one patient, cardiac arrest, and circulatory collapse at day 28 in another, and death from tumor progression in the last patient.

**Table 4.** Postoperative Complications According to Landriel-Ibanez (*n* = 79)

Grade Landriel-Ibanez classification	*N* = 79
No complication, *n* (%)	56 (70.9)
Grade 1a, no drugs, *n* (%)	5 (6.3)
Grade 1b, treatment with drugs, *n* (%)	13 (16.5)
Grade 2a, intervention without general anesthesia, *n* (%)	1 (1.3)
Grade 2b, intervention with general anesthesia, *n* (%)	0 (0.0)
Grade 3a, single organ failure treated in the intensive care unit, *n* (%)	0 (0.0)
Grade 3b, multiple organ failure treated in intensive care unit, *n* (%)	1 (1.2)
Grade 4, death, *n* (%)	3 (3.8)
Medical, *n* (%)	8 (10.1)
Surgical, *n* (%)	15 (19.0)
None, *n* (%)	56 (70.9)

**Figure 2.**
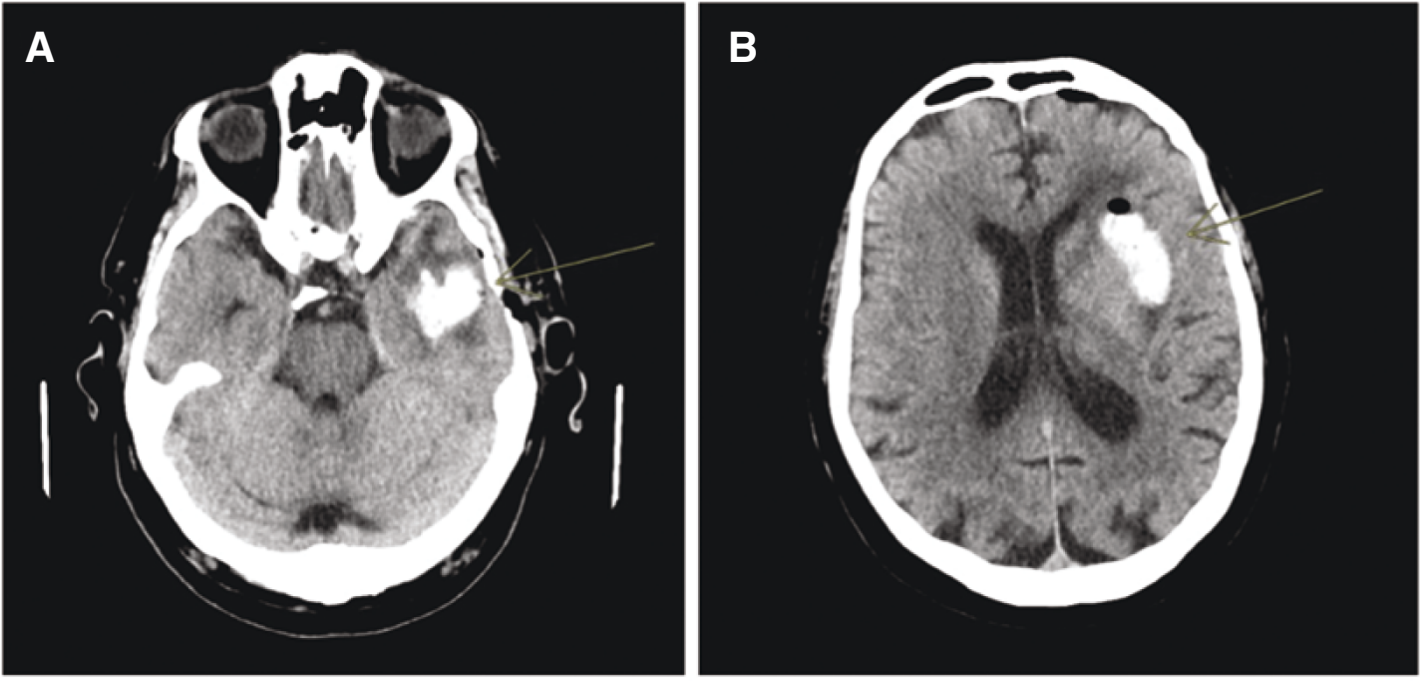
Examples of 2 postoperative hematomas on CT scan without contrast enhancement. 2A: Maximal diameter of 25 mm. 2B: Maximal diameter of 28 mm.

All but one patient (98.7%) underwent a postoperative CT scan of the 79 procedures at 6 hours after surgery as part of the clinical routine. Of these, 59.0% were negative without signs of bleeding. In 32 cases (41.0%) a bleeding was detected, although the bleeding was less than 10 mm (range 2–10 mm, mean 5.4 mm, SD 2.4) in 26 cases. In 5 patients, the hematoma was larger than 10 mm (range 15–28 mm, mean 22.6 mm, SD 5.1). None of the larger hematomas required surgical evacuation. See Figure 2 for 2 examples of postoperative hematoma.

## Discussion

In this study of the Stealth Autoguide™ by Medtronic the radiological data demonstrated a high number of multifocal and bilateral tumors, in patients with an increased burden of symptoms (cognitive changes in 47.2% and motor deficit in 37.5%). We demonstrate learning curves after the introduction without any statistical significance in the decrease of operation time during the study period and low influence depending on the neurosurgeon performing the procedure. The rate of complications and postoperative hematoma seems to be in line with current literature.^20,21^

The radiological diagnosis was correct in 62.0% of cases, slightly higher than the findings by Pennlund et al (54.6%).^4^ Diagnosis was among the suggested in 15.2% and incorrect in 10.1%. In 10 cases (12.7%) the histopathology was inconclusive. Perhaps this slightly higher rate is attributable to development of noninvasive radiological methods.^22^ Even though a radiological diagnosis of glioma can be made with high certainty, the molecular status needed to determine the appropriate treatment strategy is not yet possible to deduce from radiology alone.^23,24^ Further work with PET shows promising results for noninvasive O[6]-methylguanine-DNA methyltransferase (MGMT) status, which would be much needed in patients with non-resectable high-grade gliomas to avoid the surgical risk associated with biopsy.^25^

When introducing new surgical techniques into clinical practice, a technical learning curve is to be expected. Furthermore, robot-assisted biopsies in neurosurgery seem to be studied to a lesser extent with regard to learning aspects.^9,14^ Questions concerning the optimal ratio of surgeons to be trained and the requisite number of cases necessary for proficiency in newly introduced clinical techniques have been raised. As visualized in Figure 1 the tendency of the curve indicates a decrease in the time needed for the technique as more and more procedures are performed. However, the statistical analysis showed no significant difference regarding the improved efficiency, which may suggest that introduction in clinical practice has been fairly streamlined since the beginning. Additionally, it was found that only 12% of the variation in the duration of surgery was influenced by the surgeon, the being affected by other factors, implicating that the performing surgeon does not greatly affect the surgery duration. This finding in combination with lack of statistical significance for the decrease in the duration of a surgery over time suggests that the Stealth Autoguide™ is a safe biopsy method in clinical practice, as it does not matter which surgeon is performing the procedure. The duration of surgery in our study was comparable to previous data on the Remebot® frameless system.^26^ We found also that few biopsies (1–2) showed a longer duration of surgery (mean 60.5 min) compared to 5 or more biopsies (mean time of 42.8 min). This might reflect that few biopsies were taken in more sensitive areas where there was a higher risk of complications.

In a previous study from our department, the rate of inconclusive biopsies was 4.8% in frame-based stereotactic biopsies.^4^ The study by Mallereau et al additionally studied the diagnostic rate in previous literature and found the inconclusive biopsies to be in a range of 2.6%–11.1% in frameless biopsies compared to 0.7–15.7 in frame-based procedures.^9^ A systematic review of robot-assisted biopsied by Marcus et al from 2018 found the diagnostic rate to reside between 75% and 100% when studying one retrospective cohort and 14 case series or reports, although the size and location of studied pathologies included in the review were not specified in detail.^5^ A large case series of robot-assisted biopsies with Remebot® system by Liu et al found a diagnostic rate of 98.2%.^27^ Our rate at 87.3% diagnostic yield can perhaps result from a lower threshold to perform a robot-assisted biopsy as this is not as resource-demanding as a stereotactic procedure. Of the inconclusive 10 cases, 4 demonstrated no or mild/patchy enhancement, which is associated with a higher risk of inconclusive biopsies. Furthermore, the high rate of inconclusive biopsies from the posterior fossa may suggest that this approach perhaps carries increased inherent technical difficulties for this type of procedure,^28^ implying that these procedures perhaps warrant a more cautious selection or open biopsies to secure a conclusive histopathological diagnosis. Furthermore, 2 of the infratentorial cases were rather small targets (< 10 mm). The O-arm was used in 2 of the infratentorial cases; one was inconclusive, and perhaps this should be clinical routine. Of the inconclusive cases, PET was performed in 20%. As seen in our material, lymphomas treated with steroids pose a risk for an inconclusive biopsy and support avoiding the administration of steroids when there is a suspicion of primary CNS lymphoma prior to the biopsy procedure. Caution should be taken when planning a biopsy for suspected CNS lymphoma, and a preoperative MRI as close as possible to the biopsy operation should be routine, especially in those patients treated with steroids.

The rate of complications in our study appears comparable to similar studies,^20,21^ as well as the 30-day mortality.^29^ As for postoperative hematoma, one of the most feared intra- and postoperative complications, a CT scan without contrast was performed 6 hours after biopsy at our department as part of clinical praxis. In 39.7% of patients, a hematoma could be identified post-operatively, although it was rarely of clinical significance. As for the frame-based stereotactic biopsies performed at the same department, the number of postoperative hematomas was not studied and reported in a systematic matter; however, the single patient suffering 30-day mortality (0.8%) was due to a postoperative intracranial hemorrhage, even after hematoma evacuation.^4^ In our data 3 patients passed away within 30 days from surgery. In 2 of these, the surgery could have contributed to the cause of death (one patient suffered from epilepsy and pulmonary embolism on day 14 and the second cardiac arrest and circulatory collapse on day 28). These data support the known fact of no surgery being free from risk, especially in patients with other comorbidities as in this cohort.

For frame-based and frameless stereotactic biopsies, previous studies demonstrate a rate of hemorrhage from 28% up to 59.8% on postoperative head CT scans; the majority being smaller than 10 mm.^30–32^ For robot-assisted neurosurgical biopsies a rate of 5.77% of patients presented a postoperative hematoma on head CT, although the size of hematomas was not reported.^8^ An additional study of a robot-assisted stereotactic biopsy system identified postoperative hematomas larger than 10 mm in 8% of patients slightly higher than our 6.5%.^16^

### Strengths and Limitations

This data represents the experiences from a single clinic where the Stealth Autoguide™ technique was introduced. The strength of single-central studies is the reduced confounding factors due to the high control of variables. Since our department is the only one in the region performing neurosurgical procedures there is no selection bias or lack of surgical complications, as all these patients are referred to this department for neurosurgical treatment, such as postoperative hematomas in need of surgical evacuation. Limitations include the retrospective approach, due to the imitated access of new research questions and variables. Additionally, the quality of the collected data in retrospective studies is often limited.

## Conclusion

Robot-assisted biopsies with Stealth Autoguide™ seem to be comparable, in regard to complications, to frame-based and frameless neurosurgical biopsies. The diagnostic yield is lower compared to frame-based biopsies performed in the same department, which could indicate a lower threshold for biopsy indication. Learning curves demonstrated no statistical differences in time of surgery and only 12% surgeon-related variation (ie, variation caused by change of performing surgeon), suggesting a successful implementation of this technical adjunct with regards to complications and patient safety.
